# GCLC desuccinylation regulated by oxidative stress protects human cancer cells from ferroptosis

**DOI:** 10.1038/s41418-025-01505-8

**Published:** 2025-04-05

**Authors:** Zixiang Chen, Kaifeng Niu, Mengge Li, Yuchun Deng, Ji Zhang, Di Wei, Jiaqi Wang, Yongliang Zhao

**Affiliations:** 1 China National Center for Bioinformation, Beijing, China; 2 Beijing Institute of Genomics, Chinese Academy of Sciences, Beijing, China; 3 University of Chinese Academy of Sciences, Beijing, China

**Keywords:** Cell biology, Enzyme mechanisms

## Abstract

Tumor cells evolve strong antioxidant capacities to counteract the abnormal high level of reactive oxygen species (ROS) in the tumor microenvironment. Glutamate-cysteine ligase catalyzing subunit (GCLC) for synthesis of antioxidant glutathione (GSH) represents the key enzyme to maintain redox homeostasis of tumor cells, however, whether its activity is regulated by posttranslational modifications, such as succinylation, remains to be clarified. Here, we demonstrate the existence of succinylation modification on GCLC by in vitro and in vivo assays. NAD-dependent deacetylase Sirtuin-2 (SIRT2) serves as the desuccinylase and catalyzes GCLC desuccinylation at sites of K38, K126, and K326. Specifically, GCLC directly interacts with SIRT2, which can be substantially enhanced upon ROS treatment. This strengthened association results in GCLC desuccinylation and activation, consequently promoting GSH synthesis and rendering cancer cells resistant to ferroptosis induction. Depletion of SIRT2 decreases total GSH level and meanwhile increases the cellular susceptibility to ferroptosis, which can mostly be rescued by introducing wild-type GCLC, but not its 3K-E mutant. We further demonstrated that histone acetyltransferase P300 serves as the succinyltransferase of GCLC, and their association is remarkably decreased after ROS treatment. Thus, SIRT2-regulated GCLC succinylation represents an essential signaling axis for cancer cells to maintain their redox balance in coping with oxidative stress-induced ferroptosis.

## Introduction

Increased production of ROS is usually found in tumor cells due to the intense metabolic activities [1]. While low to moderate level of ROS acts as signal transducers participating in various biological activities, excessive accumulation of ROS without prompt and effective clearance can cause oxidative damages [2, 3]. As such, tumor cells develop multiple mechanisms to elevate their antioxidant capacities for redox homeostasis and prevention of ROS-elicited cell death [4], such as augmented activity of glucose-6-phosphate dehydrogenase (G6PD) for NAPDH generation [5], and activation of a list of transcription factors, including activator protein 1, HIF-1α, nuclear erythroid 2-related factor (NRF2), heat shock factor 1, nuclear factor kB, and tumor suppressor protein p53 [6].

Glutathione (GSH) is an endogenously synthesized multifunctional tripeptide not only participating in various biological processes, but also acting as a core antioxidant to protect cells from oxidative damages [7, 8]. GSH is the cofactor of glutathione peroxidase 4 (GPX4) responsible for reducing the cytotoxic lipid peroxides, and its depletion can result in defective antioxidant capacity and induce iron-dependent and lipid peroxidation-initiated ferroptosis [9, 10]. Cellular GSH level is maintained by a complex homeostatic mechanism, among which glutamate cysteine ligase (GCL), consisting of catalytic (GCLC) and regulatory (GCLM) subunits, plays major roles in GSH synthesis. Moreover, as a rate-limiting enzyme, GCLC catalyzing activity is subject to feedback inhibition by GSH [11], and abnormal expression is closely related to tumor progression and drug resistance in different histological types of human cancers [12, 13]. Although post-translational modification(s) (PTMs) have been proposed to directly mediate the rapid activation of GCL activity under oxidative stress [14, 15], the specific type of PTMs implicated in this process remains to be identified.

Succinylation modification is one major type of PTMs, involving the transfer of a succinyl group (-CO-CH2-CH2-CO-) from succinyl-CoA to the ε-amino group of lysine residues in target proteins [16]. Since a succinyl-group transfer introduces a relatively large structural moiety (100 kDa) compared to lysine methylation and acetylation [16], it has been proposed that succinylation modification could induce a relatively large shift in the charge and the structural alteration with significant effects on the function of the target protein. Succinylation modification regulates either protein stability or proteinase activity, and has been implicated in various biological activities such as glycolipid metabolism, amino acid metabolism, and ketone synthesis [17, 18]. However, whether GCLC activity is regulated by succinylation modification, in particular under oxidative stress in cancer cells, remains unclear.

To this purpose, in vitro and in vivo assays were employed to test the existence of succinylation modification on GCLC, and the influence on its enzymatic activity was further determined by quantificationally measuring the cellular GSH levels. Through identifying SIRT2 as the desuccinylase of GCLC, their corroborative roles in coping with the excessive oxidative stress-induced ferroptosis in cancer cells were further determined. Overall, our present study demonstrated that ROS induces GCLC desuccinylation and consequent activation through enhancing its interaction with desuccinylase SIRT2, which represents an essential mechanism for cancer cells to evade oxidative stress-induced ferroptosis, and constitutes a potential target for clinical cancer treatment.

## Results

### GCLC undergoes succinylation modification

Although PTMs have been suggested to mediate the rapid GCL activation under oxidative stress [14, 15], the underlined PTMs for this effect remain to be identified. To this aim, we examined two major types of PTMs, including acetylation and succinylation on GCLC, the major catalyzing subunit of GCL. Through transfection of Flag-tagged GCLC into HEK293T cells followed by co-immunoprecipitation (co-IP) assays, GCLC was shown to be both acetylated and succinylated (Fig. S1A). Since the addition of the succinyl group is proposed to have a more profound effect on the spatial conformation and further function of the targeted protein [19], we therefore focused on the biological significance of succinylation modification on GCLC in the following studies.

**Fig. 1:**
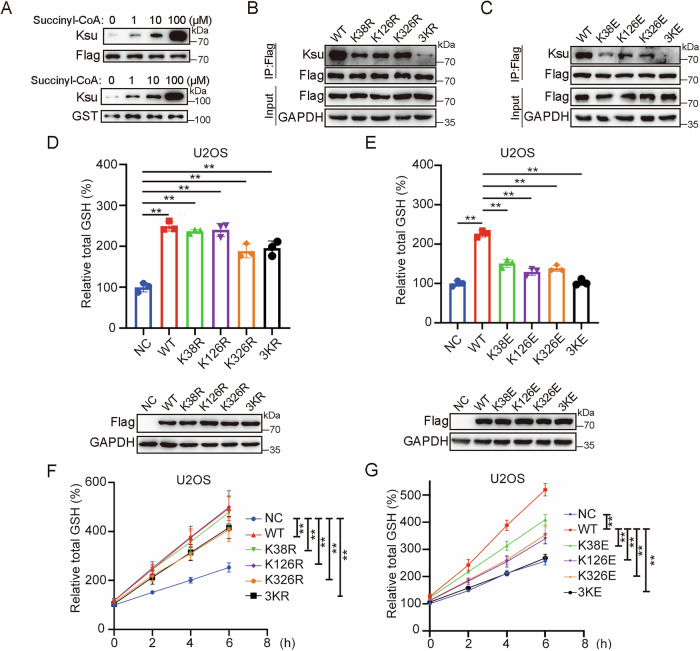
K38, K126, K326 are the major succinylation sites of GCLC, and succinylation inhibits GCLC activity. **A** In vitro GCLC succinylation assay. Flag-GCLC purified from HEK293T cells or GST-GCLC expressed in *E. coli* was incubated with different concentrations of succinyl-CoA as indicated for 4 h at 37 °C. Protein succinylation level was detected by western blotting. **B**,** C** Mapping the major lysine PTM sites of GCLC. WT GCLC and indicated K to R or K to E mutation constructs were transfected into HEK293T cells for 48 h, cell lysates were immunoprecipitated with anti-Flag antibody and analyzed by western blotting with indicated antibodies. **D** Mimicking desuccinylation of GCLC shows marginal effect on GSH synthesis. U2OS cells were transfected with indicated expression vectors for 48 h, intracellular GSH level and the expression of proteins were determined. **E** Mimicking succinylation of GCLC inhibits GSH synthesis. U2OS transfected with indicated expression constructs for 48 h, intracellular GSH level and the expression of proteins were examined. **F** Mimicking desuccinylation of GCLC shows little effect on GSH synthesis rate. U2OS cells were transfected with indicated expression vectors for 36 h, then treated with cystine free medium for 36 h followed by re-culture in complete medium, intracellular GSH level was measured at indicated time points. **G** Mimicking succinylation of GCLC inhibits GSH synthesis rate. Data were showed as mean ± standard error of mean (SEM) from at least three independent experiments. Statistical analysis by one-way or two-way ANOVA. *P  ≤  0.05, **P  ≤  0.01.

We first employed an in vitro assay to validate the succinylation level of GCLC. Purified Flag-GCLC or recombinant GST-GCLC protein was incubated with a gradient concentration of succinyl-CoA in succinylation buffer, followed by western blotting analysis using an antibody against succinylated lysine (K). The result showed that the purified GCLC undergoes succinylation modification with a gradually enhanced level following the increase of succinyl-CoA concentration (10–100 μM) (Fig. 1A).

It has been reported that sodium succinate which can be converted into succinyl-CoA by succinyl-CoA synthetase, significantly enhances the global profile of lysine succinylation [17]. We then treated Flag-GCLC overexpressed HEK293T cells with different concentrations of sodium succinate for 24 h, and the result presented a concentration-dependent enhancement in GCLC succinylation (Fig. S1B). On the contrast, depletion of succinyl-CoA obviously reduced the GCLC succinylation level in Flag-GCLC overexpressed HEK293T cells by treatment with 5 μM Fasnall [20] for 24 h (Fig. S1C). Overall, these findings suggest that the available concentration of succinyl-CoA can directly affect the succinylation level of GCLC.

### GCLC succinylation leads to a decreased enzymatic activity

To precisely locate the K sites undergoing succinylation modification, we individually mutated all forty lysine sites of GCLC to arginine (R) (mimicking the desuccinylated state). The mutated Flag-GCLC plasmid was then transfected into HEK293T cells, followed by co-IP and western blotting analysis. The result showed that mutations at sites of K38, K126, or K326 substantially decrease the succinylation level of GCLC, and simultaneously mutating all these three sites led to a nearly undetectable level of succinylation modification (Figs. 1B and S1D). We further mutated these three sites to glutamic acid (E) to mimick the negatively charged succinylation modification. Consistently, GCLC 3K-E mutant also showed a nearly absent succinylation modification (Fig. 1C). Examination of acetylation level failed to show any obvious difference between WT GCLC and its 3K-R mutant (Fig. S1E). Moreover, all these three K sites are conserved across species (Fig. S1F).

We then tried to determine whether succinylation modification on GCLC affects its catalyzing activity. As expected, overexpression of WT GCLC or its desuccinylation-mimicking mutants (K38R, K126R, K326R or 3KR) in U2OS cells significantly enhanced the cellular GSH levels compared to empty vector control (NC) (Fig. 1D). In contrast, the succinylation-mimicking mutants (K38E, K126E, K326E) only induced a slightly increased cellular GSH to an extent significantly lower than WT GCLC. In particular, cells overexpressing 3KE mutant showed a similar GSH level as NC control (Fig. 1E), suggesting that mimicking GCLC desuccinylation significantly activates its activity, while this effect is reversed when succinylation-imitating modifications were introduced in GCLC.

We further evaluated the effect of GCLC succinylation on its enzymatic activity by measuring GSH synthesis rate in U2OS cells transfected with either Flag-tagged WT GCLC or its K-R and K-E mutants. Cellular GSH was firstly depleted by culturing the cells in cystine-free medium for 36 h, and then allowed to be synthesized by re-culturing in complete medium (Fig. S2). Consistent results showed that GSH synthesis rate is significantly increased in cells overexpressing WT GCLC or its K-R mutants (K38R, K126R, K326R, 3KR) (Fig. 1F), whereas cells transfected with individual K-E (K38E, K126E, K326E) or 3KE mutant only showed a slight increase or even unchanged GSH synthesis rate in relative to NC control (Fig. 1G). These results provide further evidence that succinylation-mimicking K-E mutations nearly abolish GCLC enzymatic activity.

### Oxidative stress stimulates GSH synthesis through desuccinylation of GCLC

**Fig. 2:**
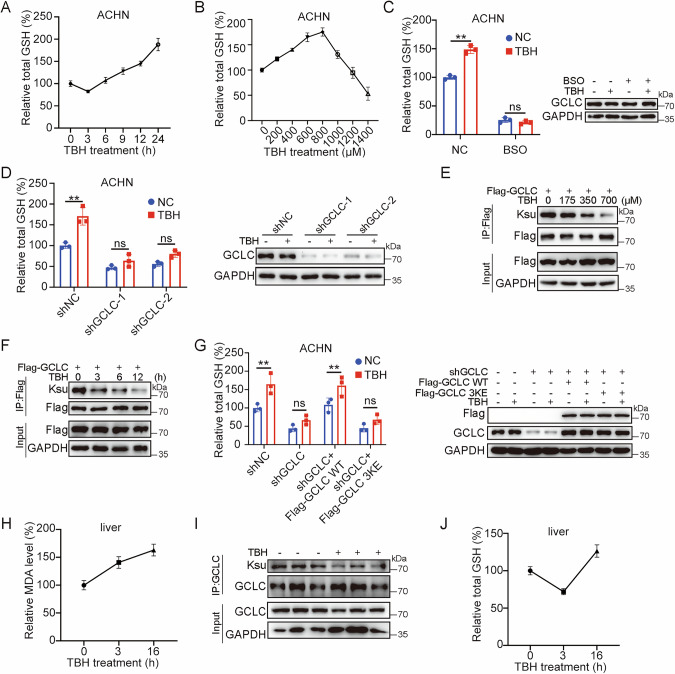
TBH increases cellular GSH level through desuccinylating GCLC. **A**, **B** TBH enhances cellular GSH. ACHN cells were treated with 700 μM TBH for different times, intracellular GSH level was measured (**A**). ACHN cells were treated with different concentrations of TBH for 24 h, intracellular GSH level was measured (**B**). **C** GCLC inhibitor BSO inhibits the increase of GSH level induced by TBH. ACHN cells were pretreated with 100 μM BSO for 12 h, then treated with 500 μM TBH for 24 h, intracellular GSH level and the expression of proteins were determined. **D** Knocking down of GCLC inhibits the increase of GSH level induced by TBH. ACHN cells were transfected with indicated expression constructs for 72 h, then treated with 700 μM TBH for 24 h, intracellular GSH level and the expression of proteins were examined. **E** TBH decreases GCLC succinylation in a dose-dependent manner. U2OS cells with stable overexpression of Flag-GCLC were treated with different concentrations of TBH as indicated for 12 h. Cell lysates were immunoprecipitated with anti-Flag and analyzed by western blotting with indicated antibodies. **F** TBH decreases GCLC succinylation level in a time-dependent manner. Flag-GCLC overexpressing U2OS cells were treated with 700 μM TBH for different times. Cell lysates were immunoprecipitated with anti-Flag and analyzed by western blotting using indicated antibodies. **G** Succinylation-mimicking GCLC inhibits the increase of GSH level induced by TBH. ACHN cells were transfected with indicated expression constructs for 72 h, then treated with 700 μM TBH for 24 h, intracellular GSH level and the expression of proteins were analyzed. **H** TBH promotes MDA level in mouse liver tissue. TBH (300 mg/kg) was intraperitoneal injected into mice for 3 h or 16 h (*n* = 5). Liver tissues were harvested and MDA was measured using lipid peroxidation MDA assay kit. **I** TBH decreases GCLC succinylation in vivo. TBH (300 mg/kg) was intraperitoneal injected into mice for 16 h (*n* = 3). Liver tissues lysates were immunoprecipitated with anti-GCLC and analyzed by western blotting with indicated antibodies. **J** TBH enhances cellular GSH in vivo. TBH (300 mg/kg) was intraperitoneal injected into mice for 3 h or 16 h (*n* = 5). Liver tissues were harvested and total GSH was measured using total glutathione assay kit. Data were showed as mean ± standard error of mean (SEM) from at least three independent experiments. Statistical analysis by one-way or two-way ANOVA. *P  ≤  0.05, **P  ≤  0.01.

We first determined the regulatory role of GCLC in GSH synthesis under oxidative stress. Human renal cell carcinoma (RCC) cells ACHN were treated with 700 μM TBH, and the results showed that cellular GSH level was decreased at 3 h post treatment, but rapidly increased thereafter (Fig. 2A), suggesting that oxidative stress leads to exhaustion of cellular GSH at earlier treatment which could be rapidly compensated through activated GCLC activity. Similarly, a dose-dependent increase of GSH level was also observed at 200–800 μM of TBH treatment for 24 h, whereas higher dose (>1000 μM) resulted in a rapid decrease of GSH level owing to significantly increased cell death (Fig. 2B). Similar results were also obtained in U2OS and human RCC line CAKI-1 (Fig. S3A–D). Moreover, functional inhibition of GCLC in ACHN cells by GCLC inhibitor BSO or shRNA significantly inhibited the GSH synthesis induced by TBH (Fig. 2C, D). These results suggest a GCLC-dependent GSH synthesis under oxidative stress.

We then tested whether oxidative stress regulates GCLC succinylation level. U2OS cells with stably overexpressing Flag-GCLC were treated with TBH or H_2_O_2_. The results showed a significant decrease in GCLC succinylation level upon both treatments, with dose- and time-dependent manners (Figs. 2E, F and S3E).

We further explore the potential significance of GCLC succinylation in oxidative stress-modulated GSH synthesis. GCLC-depleted ACHN cells were reconstituted with either WT GCLC or its succinylation-mimicking 3KE mutant, followed by the treatment of 700 μM TBH for 24 h. The result demonstrated that GSH synthesis capacity in WT GCLC-reconstituted cells is nearly recovered to the control level of shNC, but significantly inhibited in GCLC 3KE cells (Fig. 2G). This data strongly suggests that oxidative stress-stimulated GSH synthesis depends on GCLC and its desuccinylation status.

We further examined whether oxidative stress regulate GCLC succinylation and GSH synthesis in vivo. C57BJ/6 male mice with 8-week old were divided into three groups (each containing 5 mice), and subjected to the TBH treatment by intraperitoneally injecting TBH at dose of 300 mg/kg. Liver tissues, where the GSH is mainly synthesized [21], were then isolated from the treated mice at 0, 3, and 16 h. The MDA (malondialdehyde) levels were firstly measured by lipid peroxidation assay kit, and the result showed that relative MDA level was progressively increased upon prolonged TBH treatment, indicating that the liver tissue did suffer from oxidative damage (Fig. 2H). Then, the endogenous GCLC was enriched by anti-GCLC antibody, and western blotting result showed that TBH treatment for 16 h substantially reduces the in vivo succinylation level of GCLC (Fig. 2I). Furthermore, GSH level in liver tissue presented a sharp decrease at 3 h, followed by a significant enhancement at 16 h post treatment (Fig. 2J). This change tendency in both GCLC succinylation and GSH synthesis showed a similar pattern as the one in culturing cells, strongly supporting that oxidative damage induces GCLC desuccinylation and promotes GSH synthesis in vivo.

### SIRT2 serves as the desuccinylase of GCLC

**Fig. 3:**
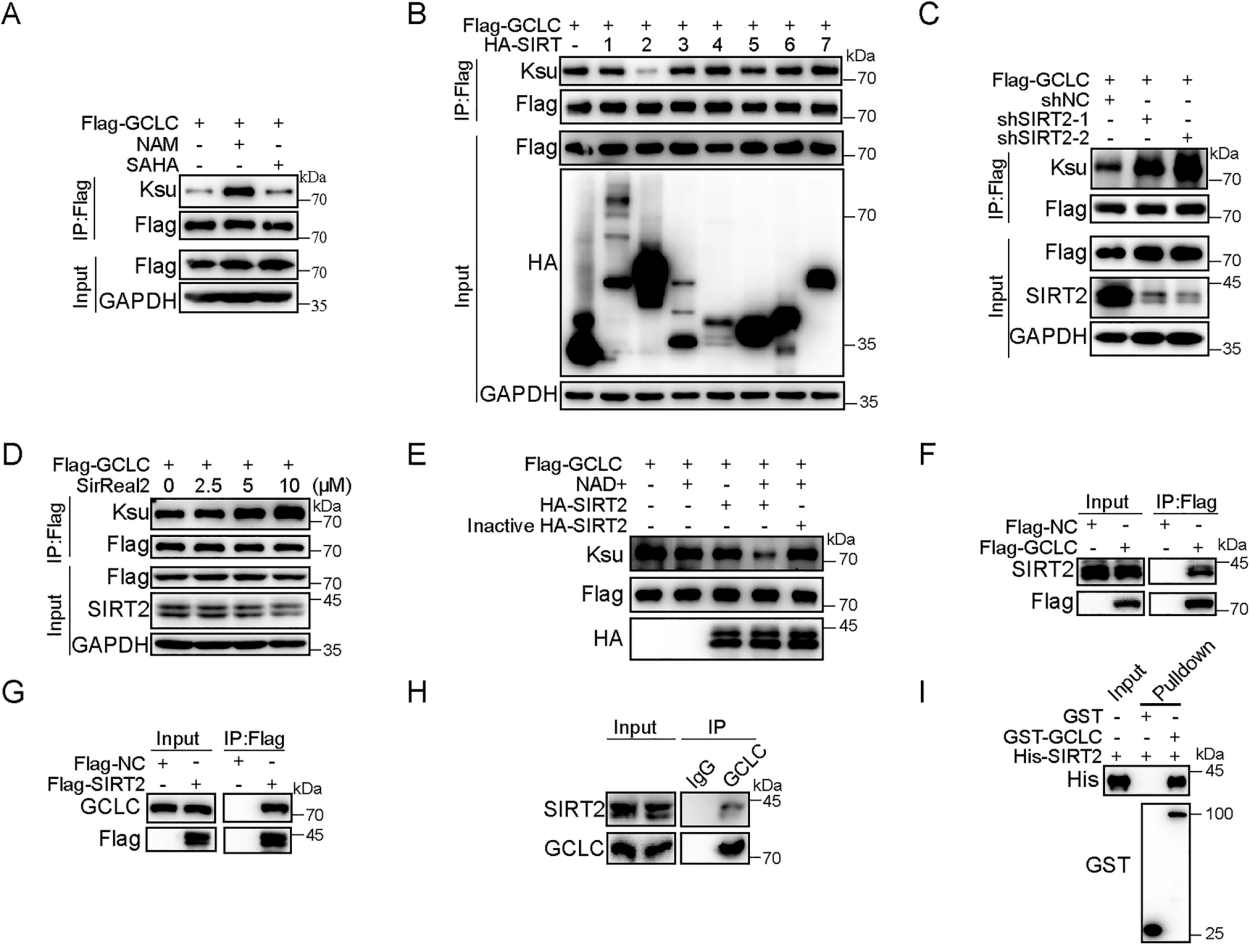
SIRT2 desuccinylates GCLC by directly interacting with GCLC. **A** GCLC succinylation is regulated by HDAC III. HEK293T cells were transfected with Flag-GCLC for 36 h, then treated with DMSO, nicotinamide (NAM, 20 mM) or Vorinostat (SAHA, 10 μM) for 24 h. Cell lysates were immunoprecipitated with anti-Flag and analyzed by western blotting. **B** SIRT2 desuccinylates GCLC. HEK293T cells were transfected with indicated expression constructs for 48 h, followed by immunoprecipitation with anti-Flag and western blotting analysis. **C** Knocking down of SIRT2 enhances the succinylation of GCLC. U2OS cells were transfected with indicated expression constructs for 72 h, followed by immunoprecipitation and western blotting analysis. **D** SIRT2 inhibitor SirReal2 enhances the succinylation of GCLC. HEK293T cells were transfected with Flag-GCLC for 36 h, then treated with different concentrations of SirReal2 as indicated for 24 h. Cell lysates were immunoprecipitated with anti-Flag and analyzed by western blotting with indicated antibodies. **E** SIRT2 desuccinylates GCLC in vitro. Purified GCLC proteins were desuccinylated in the presence of purified SIRT2 in vitro. See “Methods” for further details. After in vitro desuccinylation reaction, samples were analyzed by western blotting with indicated antibodies. **F** Flag-GCLC interacts with endogenous SIRT2. **G** Flag-SIRT2 interacts with endogenous GCLC. **H** Endogenous association between GCLC and SIRT2. Whole-cell lysates were immunoprecipitated with control IgG or anti-GCLC antibodies, and the pull-down protein complex was analyzed by western blotting. **I** GCLC interacts with SIRT2 in vitro. Purified GCLC proteins were incubated with SIRT2 in vitro. See “Methods” for further details. Samples were analyzed by western blotting with indicated antibodies.

Previous studies reported that protein desuccinylation is regulated by some histone deacetylase (HDAC) family members, including class III sirtuin 5 and 7 (SIRT5 and SIRT7) and class I HDAC1-3 [22–24]. The findings that GCLC desuccinylation activates its enzymatic activity and subsequent GSH synthesis prompted us to search for the responsible desuccinylase. HEK293T cells overexpressing Flag-GCLC were treated with class I and II HDAC inhibitor Vorinostat (SAHA) or class III inhibitor nicotiamide (NAM). The result showed that GCLC succinylation level was markedly increased in HEK293T cells with NAM but not with SAHA, suggesting that GCLC desuccinylation was regulated by class III HDACs (Figs. 3A and S4A). Then, each of class III HDACs family members (SIRT1-7) was co-expressed with Flag-GCLC in HEK293T cells. The results demonstrated that only overexpressing SIRT2 could substantially decrease the succinylation level of GCLC (Fig. 3B). Specifically, overexpressing SIRT2 didn’t affect the acetylation level of GCLC (Fig. S4B). To further validate the above findings, SIRT2 was silenced by shRNA or inhibitor SirReal2 in U2OS cells, and the result showed that SIRT2 silencing remarkably enhances the succinylation level of GCLC compared to their controls (Fig. 3C, D). Further results from in vitro desuccinylation assays also demonstrated that HA-SIRT2 protein purified from HA-SIRT2 overexpressing HEK293T cells could efficiently desuccinylate GCLC in the presence of NAD^+^ (Fig. 3E), while inactivated HA-SIRT2 protein (95 °C for 10 min) showed a total loss of desuccinylase activity. Overall, these findings firmly support SIRT2 as the desuccinylase of GCLC.

### SIRT2 directly interacts with GCLC

As SIRT2 could desuccinylate GCLC, we then explored the physical association between SIRT2 and GCLC, especially under oxidative stress condition. Through co-IP assays, endogenous SIRT2 was detected with anti-SIRT2 antibody in the Flag-GCLC pull-down complex (Fig. 3F). Likewise, endogenous GCLC was also observed in the Flag-SIRT2 immunoprecipitated complex with anti-GCLC antibody (Fig. 3G). To further establish their endogenous associations, HEK293T whole cell lysates were incubated with control IgG or anti-GCLC antibody, followed by western blotting analysis on the pull-down complex. Endogenous SIRT2 was found to be co-precipitated with GCLC, but not with control IgG (Fig. 3H). Moreover, a direct interaction between SIRT2 and GCLC was further confirmed by an in vitro glutathione S-transferase (GST) pull-down assay (Fig. 3I). Nevertheless, truncated SIRT2-based interaction assays showed that GCLC only associates with the catalytic core domain, instead of N- and C-terminus, of SIRT2 (Fig. S4C). Thus, our findings provide strong evidence that SIRT2 directly interacts with GCLC.

### SIRT2 regulates GCLC-dependent GSH synthesis

**Fig. 4:**
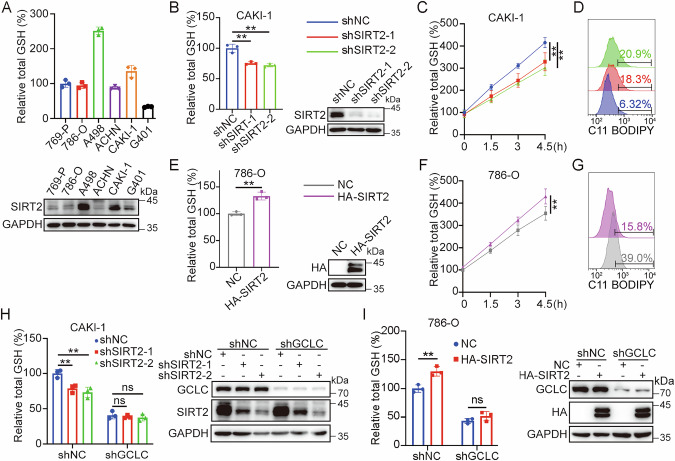
SIRT2-regulated GSH synthesis depends on GCLC. **A** Well-correlated expression between intracellular GSH level and SIRT2 in RCC cell lines. Intracellular GSH level and the expression of SIRT2 were measured in 6 different RCC cell lines. **B**–**D** Knocking down of SIRT2 decreases cellular GSH synthesis and enhances lipid peroxidation. CAKI-1 cells were transfected with indicated expression constructs for 72 h, intracellular GSH level and the expression of proteins were examined (**B**). CAKI-1 cells were transfected with indicated expression constructs for 36 h, then treatment with cystine free medium for 36 h followed by re-culture in complete medium. Intracellular GSH level was measured at indicated time points (**C**). CAKI-1 cells were transfected with indicated expression constructs for 72 h, lipid peroxidation level was examined (**D**). **E**–**G** Overexpression of SIRT2 enhances cellular GSH synthesis and decreases lipid peroxidation. CAKI-1 cells were transfected with indicated expression constructs for 48 h, intracellular GSH level and the expression of proteins (**E**), GSH synthesis rates (**F**), and lipid peroxidation level (**G**) were measured. **H** Knocking down of SIRT2 didn’t affect cellular GSH level in GCLC knockdown CAKI-1 cells. Indicated expression constructs were transfected into control or GCLC knockdown CAKI-1 cells for 72 h, intracellular GSH level and the expression of proteins were examined. **I** Overexpression of SIRT2 didn’t affect the cellular GSH level in GCLC knockdown 786-O cells. Data were showed as mean ± standard error of mean (SEM) from at least three independent experiments. Statistical analysis by Student’s *t* test, one-way or two-way ANOVA. *P  ≤  0.05, **P  ≤  0.01.

As SIRT2 regulates GCLC succinylation level, its regulatory role in GSH synthesis was further determined. We first examined the SIRT2 expression and GSH levels in a list of human RCC cell lines, and observed a well-correlated expression pattern (Fig. 4A), with high GSH level corresponding to higher SIRT2 expression (CAKI-1, A498) versus low GSH with lower SIRT2 expression (769-P, 786-O, ACHN, G401). SIRT2 expression was then knocked down by shRNA in SIRT2-high cell lines (CAKI-1 and A498), and cellular GSH level were found to be significantly reduced (Figs. 4B and S5A). GSH synthesis rate was also significantly decreased post SIRT2 knockdown (Fig. 4C). Further treatment with SIRT2 inhibitor SirReal2 led to a dose-dependent decrease in cellular GSH level (Fig. S5B). These results suggest a critical role of SIRT2 in regulating cellular GSH level.

It is well established that GSH serves as the reducing substrate of GPX4 activity to scavenge toxic oxidative metabolites [25]. As expected, SIRT2 knockdown in CAKI-1 cells increased cellular ROS and lipid peroxidative levels (Figs. 4D and S5C). In contrast, overexpressing SIRT2 in SIRT2-low cell lines (786-O, ACHN) markedly enhanced cellular GSH level and synthesis rate (Figs. 4E,F and S5D), while ROS and lipid peroxidative levels were significantly decreased (Figs. 4G and S5E). These results demonstrate a critical role of SIRT2 in regulating cellular GSH and anti-oxidative capacities.

To determine whether SIRT2-regulated GSH synthesis depends on GCLC, we depleted SIRT2 by shRNA in GCLC knockdown CAKI-1 cells, and found that SIRT2 silencing decreases cellular GSH level in control cells but not in GCLC knockdown cells (Fig. 4H). In contrast, overexpressing SIRT2 enhanced cellular GSH level in 786-O control cells but not in GCLC knockdown 786-O cells (Fig. 4I), supporting that SIRT2-regulated GSH synthesis depends on GCLC.

### Oxidative stress activates GCLC activity dependent of SIRT2

**Fig. 5:**
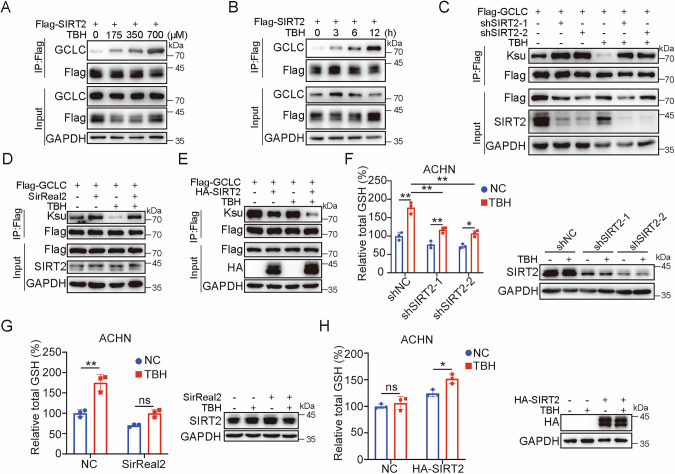
SIRT2 is essential for GCLC desuccinylation in respone to oxidative stress. **A** TBH increases the interaction between SIRT2 and GCLC in a dose-dependent manner. HEK293T cells were transfected with Flag-SIRT2 for 48 h, then treated with different concentrations of TBH as indicated for 12 h. Cell lysates were immunoprecipitated with anti-Flag and analyzed by western blotting with indicated antibodies. **B** TBH increases the interaction between SIRT2 and GCLC in a time-dependent manner. HEK293T cells were transfected with Flag-SIRT2 for 48 h, then treated with 700 μM TBH for different times as indicated. Cell lysates were immunoprecipitated with anti-Flag and analyzed by western blotting with indicated antibodies. **C** Knocking down of SIRT2 inhibits GCLC desuccinylation induced by TBH. U2OS cells with stably overexpressing Flag-GCLC were transfected with indicated vectors for 72 h, then treated with 700 μM TBH for 12 h. Cell lysates were immunoprecipitated with anti-Flag and analyzed by western blotting with indicated antibodies. **D** SirReal2 inhibits GCLC desuccinylation induced by TBH. U2OS cells with stably overexpressing Flag-GCLC were pretreated with 10 μM SirReal2, then treated with 700 μM TBH for 12 h, followed by immunoprecipitation with anti-Flag and western blotting analysis. **E** SIRT2 promotes GCLC desuccinylation induced by TBH. U2OS cells with stably overexpressing Flag-GCLC were transfected with indicated expression constructs for 48 h, then treated with 700 μM TBH for 4 h. Cell lysates were subjected to immunoprecipitation with anti-Flag and western blotting analysis with indicated antibodies. **F** Knocking down of SIRT2 inhibits the increase of GSH level induced by TBH. ACHN cells were transfected with indicated expression constructs for 72 h, then treated with 700 μM TBH for 24 h, intracellular GSH level and the expression of proteins were examined. **G** SirReal2 inhibits the increase of GSH level induced by TBH. ACHN cells were pretreated with 10 μM SirReal2 for 12 h, then treated with 700 μM TBH for 24 h, intracellular GSH level and the expression of proteins were examined. **H** SIRT2 promotes the increase of GSH level induced by TBH. ACHN cells were transfected with indicated expression constructs for 72 h, then treated with 700 μM TBH for 24 h, intracellular GSH level and the expression of proteins were measured. Data were showed as mean ± standard error of mean (SEM) of at least three independent experiments. Statistical analysis by two-way ANOVA. *P  ≤  0.05, **P  ≤  0.01.

To determine if oxidative stress-induced GCLC desuccinylation depends on SIRT2, we first tested the association between SIRT2 and GCLC under oxidative stress. The co-IP results showed that endogenous GCLC level in Flag-SIRT2 immunoprecipited complex were substantially increased in dose- and time-dependent manners after TBH treatment (Fig. 5A, B), suggesting that oxidative stress obviously promotes interaction between SIRT2 and GCLC, which might represent the major mechanism for GCLC desuccinylation in response to oxidative stress.

To validate this hypothesis, SIRT2 activity was suppressed by shRNA or inhibitor SirReal2 in U2OS cells stably overexpressing Flag-GCLC, followed by the treatment with 700 μM TBH for 12 h. The results showed that SIRT2 inhibition markedly enhances the succinylation level of GCLC even without TBH treatment. More importantly, TBH-induced decrease in GCLC succinylation level was mostly reversed by SIRT2 silencing (Fig. 5C, D). Conversely, SIRT2 overexpression decreased the GCLC succinylation level, which was more pronounced when combined with TBH treatment for 4 h (Fig. 5E). These results clearly demonstrate that SIRT2 directly desuccinylates and activates GCLC upon oxidative stress. In consistent with the activated status of GCLC regulated by SIRT2, silencing of SIRT2 by shRNA or inhibitor SirReal2 in ACHN cells followed by the treatment with 700 μM TBH led to a significantly compromised induction of GSH level (40% vs. 77% of control) (Fig. 5F, G), whereas overexpression of SIRT2 rapidly increased cellular GSH level after oxidative stress for 6 h (Fig. 5H). These data support that SIRT2 participates in the regulation of oxidative stress-induced GSH synthesis through modulating succinylation level of GCLC.

### Histone acetyltransferase P300 interacts with and succinylates GCLC

To search for the succinyltransferase of GCLC, we employed tandem affinity purification coupled with mass spectrometry analysis on lysates of Huh7 cells ectopically expressing Flag-GCLC. The histone acetyltransferase P300 with succinyltransferase activity was discovered in the list of GCLC-interacting partners (Fig. S6A). We then used co-IP assay to validate their association. Endogenous GCLC was detected with anti-GCLC antibody in the HA-P300 pull-down complex (Fig. S6B), and likewise, P300 was identified in the GCLC-pull down complex (Fig. S6C), supporting that GCLC interacts with P300. Then, HA-P300 was co-expressed with Flag-GCLC WT or 3KR in HEK293T cells. Western blotting analysis on co-IP samples demonstrated that overexpressing P300 increases the succinylation level of WT GCLC rather than its 3KR mutant (Fig. S6D), suggesting that P300 is the main succinyltransferase of GCLC. In support, the succinylation level of GCLC could be substantially reduced by treatment with P300 inhibitor (SGC-CBP30) (Fig. S6E).

We then tested whether their association is affected by ROS treatment. HEK293T cells were transfected with HA-P300 for 24 h, and then treated with 700 μM TBH for 12 h. Western blotting analysis showed that upon ROS treatment, association between GCLC and P300 was substantially decreased, in contrast to a remarkably enhanced interaction between GCLC and SIRT2 (Fig. S6F).

### GCLC desuccinylation protects cancer cells from ferroptosis

**Fig. 6:**
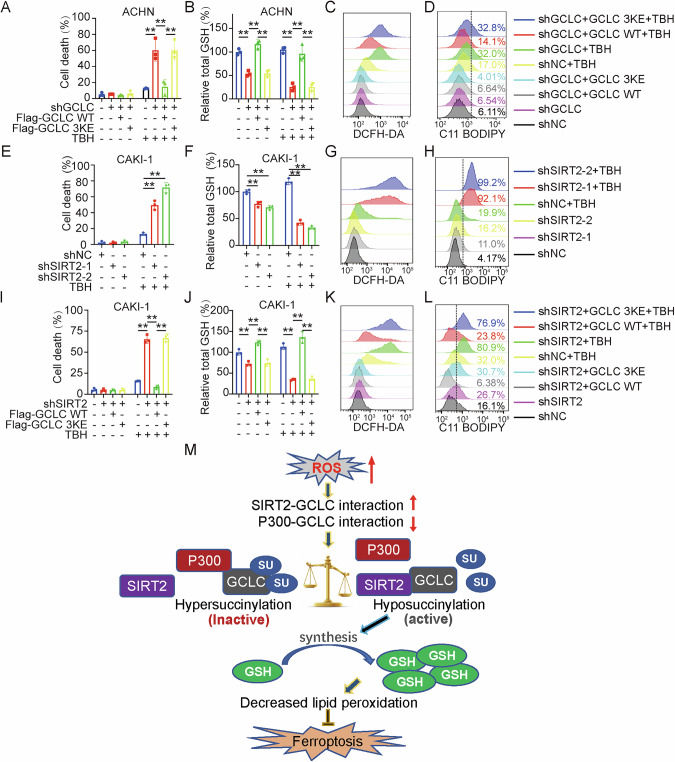
SIRT2 regulates TBH-induced ferroptosis through desuccinylating GCLC. **A**–**D** Succinylation of GCLC enhances TBH-induced ferroptosis. Indicated cells were treated with 1000 µM TBH for 12 h, and cell death by PI staining coupled with flow cytometry (**A**) and intracellular GSH level (**B**) were examined. Cells with 4 h of treatment with 1000 µM TBH were used to determine the ROS level by flow cytometry using DCFH-DA (**C**) and the lipid peroxidation level by flow cytometry using C11-BODIPY (**D**). **E**–**H** Knocking down of SIRT2 enhances TBH-induced ferroptosis. Indicated cells were treated with 2400 µM TBH for 12 h. Cell death by PI staining coupled with flow cytometry (**E**) and intracellular GSH level (**F**) were determined. Cells with treatment of 2400 µM TBH for 4 h were used to determine the ROS level by flow cytometry using DCFH-DA (**G**) and the lipid peroxidation level by flow cytometry using C11-BODIPY (**H**). **I**–**L** SIRT2 depletion-induced ferroptosis can be rescued by overexpression of WT GCLC but not the 3KE mutant. Cell treatments were same as the above. **M** Oxidative stress regulates sensitivity to ferroptosis through SIRT2-GCLC-GSH axis. GCLC is succinylated at K38, K126, K326, leading to inhibited GCLC activity. Oxidative stress enhances the direct interaction of GCLC with desuccinylase SIRT2, resulting in GCLC de-succinylation and activated enzymatic activity, which promotes GSH synthesis and protects the cancer cells from oxidative stress induced ferroptosis. Data were showed as mean ± standard error of mean (SEM) from at least three independent experiments. Statistical analysis by two-way ANOVA. *P  ≤  0.05, **P  ≤  0.01.

Our findings that ferroptosis inhibitor Fer-1, instead of other cell death inhibitors (apoptosis, necroptosis), could efficiently protect cells from TBH-induced cell death in SIRT2-knocked down CAKI-1 cells (Fig. S7A), suggest that SIRT2-GCLC-GSH axis may play pivotal roles in regulating sensitivity to ferroptosis in cancer cells suffering from oxidative stress. We first validated the role of GCLC succinylation in ferroptosis by inactivating GCLC with shRNA or BSO in ACHN cells. A significant augmentation in TBH-induced ferroptosis with feature of GSH exhaustion was observed (Fig. S7B–E). More importantly, this enhanced TBH sensitivity could be abolished by re-expression of shRNA-insensitive WT GCLC gene (Figs. 6A–D and S7F), but not 3KE mutant, suggesting that GCLC succinylation level largely affects its enzymatic activity and further sensitivity of cancer cells to ferroptosis induction.

We then examined the functional role of SIRT2 in regulating ferroptosis. Suppression of SIRT2 by specific shRNA or promoted TBH-induced ferroptosis in SIRT2-high CAKI-1 cells (Fig. 6E), with features of GSH depletion, ROS production, and lipid peroxidative accumulation (Figs. 6F–H and S8A). An enhanced ferroptosis were also observed by treatment with SIRT2 inhibitor SirReal2, or another ferroptosis activator RSL3 (Fig. S8B–E), pointing to the potential significance of SIRT2 in regulating ferroptosis sensitivity.

The critical role of SIRT2-mediated GCLC desuccinylation in ferroptosis was further determined. CAKI-1 cells were first silenced with SIRT2 using SIRT2 shRNA, followed by transfection of WT GCLC or its 3KE mutant. The results demonstrated that overexpression of WT GCLC but not 3KE mutant significantly rescues TBH-induced ferroptotic cell death in SIRT2-depleted cells (Figs. 6I–L and S8F). These evidences support the notion that cancer cells could avoid oxidative stress-induced ferroptosis through activating the SIRT2-GCLC-GSH signaling axis.

## Discussion

Higher level of ROS production is one of typical characteristics in tumor microenvironment, but excessive ROS are cytotoxic and can trigger senescence, apoptosis, or ferroptosis [26]. GSH is one of the most important antioxidant substances, and its active synthesis plays crucial roles in eliminating the detrimental level of ROS [4]. GSH biosynthesis is transcriptionally controlled by NRF2, one critical oxidative stress-induced transcription factor being capable of promoting transcription of multiple antioxidant genes, including GCL(GCLC/GCLM), glutathione synthetase (GSS), and a subunit of the cystine/glutamate transporter xCT (SLC7A11) indispensable for GSH synthesis, as well as other redox enzymes utilizing GSH or NADPH to reduce the oxidized substrates, such as GPX4 [27, 28]. In addition, RNA methyltransferase-mediated mRNA methylations were also shown to regulate both SLC7A11 and GPX4 expressions, and subsequent sensitivity to oxidative stress [29, 30]. Although the relative level of GCL subunits is transcriptionally regulated, oxidative stress appears to enhance cellular GCL activity via direct PTM of likely one or both GCL subunits [21]. In particular, subtoxic concentrations of oxidative agents lead to the transient activation of GCL activity without detectable increases in GCL subunit protein level [11, 31]. In support, our findings demonstrated the existence of novel succinylation modifications on GCLC, and moreover, oxidative stress can induce GCLC desuccinylation through enhancing *vs*. decreasing its association with desuccinylase SIRT2 and succinyltransferase P300, respectively, leading to a fast GCLC activation and consequently increased GSH synthesis, which endows cancer cells with phenotype of resistance to ferroptosis under oxidative stress condition (Fig. 6M).

Lysine succinylation is a naturally occurring PTM that affects the stability and function of proteins, and has been shown to play critical roles in redox homeostasis [18]. Glutaminase (GLS) succinylation increases glutaminolysis and the production of NADPH and GSH, thereby counteracting oxidative stress [32]. Succinylation of KEAP1 promotes NRF2 activation in coping with the perturbations of TCA cycle [33]. IDH2 succinylation inhibits its activity leading to increasing cellular susceptibility to oxidative stress [34]. Here, we demonstrated the existence of succinylation modification on three K sites of GCLC, and their succinylation-simulating mutations nearly abolished the GCLC catalyzing activity, suggesting a pivotal role of succinylation modification in modulating GCLC enzymatic activity.

Both acetylation and succinylation modifications can occur on the lysine sites of target proteins, and removal of both modifications is mainly processed by HDAC family [35]. Up to now, SIRT5/7 and HDAC1-3 of HDAC class III have been reported to function as a desuccinylase in addition to their deacetylation activity [22–24]. SIRT5 is a highly efficient protein lysine desuccinylase, and has been shown to participates in carcinogenic process through desuccinylating multiple targets, such as mitochondrial malic enzyme 2 (ME2) [36], pyruvate kinase M2 (PKM2) [37], serine hydroxymethyltransferase 2 (SHMT2) [38], etc. Sirt7 serves as a histone desuccinylase catalyzing H3K122 desuccinylation and functioning in DNA damage response and genome stability [23]. Moreover, ectopic expression of HDAC1/2/3 downregulated global histone succinylation and acetylation [24]. SIRT2, as one of the HDACs, has been shown to possess multiple deatylation enzyme activity, including deaceylation [35], decrotonylation [39], demethacrylation [40] and delactylation [41], and regulate a variety of biological processes, such as suppressing glycolysis [42], maintaining redox balance [43], and promoting cell cycling and division [44]. But there is no report so far about its desuccinylation enzyme activity. Our findings established the critical roles of SIRT2 in regulating redox balance through its desuccinylase activity on GCLC. Notably, even though acetylation modification exists on GCLC, its level is not affected by manipulating SIRT2 expression. Additionally, the acetylation inactive mutant of SIRT2 (H187Y) [26] is still capable of desuccinylating GCLC (Fig. S4D), suggesting that in response to oxidative stress, SIRT2 regulates GCLC activation mainly through its desuccinylation activity.

It is well established that upon oxidative stress, the cysteines of target proteins undergo oxidative PTM by a reversible oxidize of thiol groups, such as S-glutathionylation, S-nitrosylation, etc., through which numerous enzymes were regulated not only for their biological activity, but also for their cellular localization or interactions with binding partners [45]. Oxidative PTMs have been observed on SIRT family members, including SIRT2 [45], and moreover, ROS facilitate the interaction of SIRT2 with its downstream substrates, including SMC1A [46], G6PD [47], PGAM2 [48]. Therefore, it can be speculated that the change of oxidative PTM on SIRT2 under oxidative stress is most likely responsible for the enhanced association between SIRT2 and its target of GCLC.

Both enzyme-dependent and independent mechanisms have been reported to participate in the regulation of succinylation modification of substrates [17]. The non-enzymatic succinylation is mainly mediated by intracellular succinyl-CoA which can be modulated by succinyl-CoA metabolism-targeting enzymes, such as OXCT1, a member of the CoA transferase family I [49], and SUCLA2/SUCLG2, the ADP-forming succinyl-CoA synthetase [32, 50]. Meanwhile, several enzymes including carnitine palmityl transferase (CPT1A) and histone acetyltransferase family members (HAT1, KAT2A, P300) have been shown to possess succinyltransferase activity responsible for the enzymatic succinylation of target proteins [18]. In support, our in vitro result proved the existence of succinyl-CoA concentration-dependent succinylation on GCLC. Moreover, we further identified P300 as the main succinylation-catalyzing enzyme of GCLC, and their association showed a remarkable decrease upon ROS treatment. Therefore, although both nonenzymatic and enzymatic succinylations occur on GCLC, the latter mediated by P300, in cooperation with SIRT2, may play a major role in dynamically modulating the succinylation level of GCLC under stress condition.

Accumulative evidence has demonstrated the close association between increased GSH level and the resistance to chemotherapies, whereas impairment of GSH antioxidant defense system could initiate multiple forms of programmed cell death including ferroptosis in cancers [51, 52]. Since some drug-resistant tumors are more sensitive to lipid peroxidation [53], interfering the major cellular antioxidant axis of system Xc^-^-GCL-GPX4 has been extensively tested as an attractive combined strategy with chemotherapeutic agents for the treatment of drug-resistant solid tumors [54]. The ferroptosis inducers include inhibitors targeting system Xc^-^ (sorafenib) [55] and GPX4 (RSL3) [56], with ability to induce the accumulation of lipid peroxides [57], and directly suppressing GCL (L-buthionine sulfoximine, BSO) leading to GSH depletion [58]. However, these inhibitors are mainly small molecules that may be lack of tumor cell selectivity, and have the potential risk to deteriorate the side effects of the current anti-tumor drugs [59]. Since post-translational control mechanisms have been proposed to participate in the regulation of rapid oxidative activation of GCL [15, 60], inducing ferroptosis by targeting PTMs might be a promising therapeutic strategy for cancer treatment. However, few studies have investigated the post-translational regulation of GCL activity. GCLC phosphorylation has been reported, but with the effect of suppressing GCL activity [61]. Our findings are the first to demonstrate that GCLC desuccinylation mediated by SIRT2 directly regulates the rapid activation of GCLC upon oxidative stress, which provides a promising adjunct therapeutic target for cancer treatment. In particular, inhibitors specifically designed to interfere SIRT2-GCLC association and subsequent GCLC activation may be utilized to enhance tumor treatment efficacy while decreasing the toxicity to normal cells, which has the potential to ultimately improve the prognosis of cancer patients.

## Materials and methods

### Cell lines

The cell lines of 786-O, 769-P, A498, ACHN, CAKI-1, G401, U2OS, and HEK293T cells were obtained from the American Type Culture Collection. Huh7 was obtained from the Cell Resource Center, Chinese Academy of Medical Sciences. Cells were cultured in DMEM supplemented with 10% (v/v) FBS (B118-500, Nobimpex), 100 units/ml penicillin, and 100 μg/ml streptomycin (15140122, Gibco) in a humidified incubator at 37 °C with 5% CO_2_. All cell lines were tested as negative for mycoplasma contamination.

### Chemicals and antibodies

The commercial sources of chemical reagents were as follows: TBH (75-91-2, Sigma-Aldrich), RSL3 (19288, Cayman), SirReal2 (S7845, Selleck), Ferrostatin 1 (ab146169, Abcam), Z-VAD-FMK (S7023, Selleck), Necrostatin-1 (S8037, Selleck), Succinyl-CoA (S1129, Sigma-Aldrich), Nicotinamide (98-92-0, Sigma-Aldrich), Vorinostat (SC0231, Beyotime), NAD+ (S2518, Sellect), C11 BODIPY 581/591 (27086, Cayman), BSO (B2515, Sigma-Aldrich), SGC-CBP30 (S7256, Sellect), Fasnall (F413416, Aladdin). Sources of antibodies: anti-GCLC (ab190685, Abcam), anti-SIRT2 (66410-1-Ig, Proteintech), anti-SIRT2 (ab51023, Abcam), anti-HA tag (51064-2-AP, Proteintech), anti-Flag tag (20543-1-AP, Proteintech), anti-GAPDH (MAB374, Sigma-Aldrich), anti-pan-Succinyllysine (PTM-419, PTM BIO), anti-pan-acetyllysine (PTM-101, PTM BIO), anti-GST tag (10000-0-AP, Proteintech), anti-His tag (66005-1-Ig, Proteintech), anti-P300 (83078-5-RR, Proteintech).

### Lentiviral-mediated shRNA interference

All shRNAs for GCLC and SIRT2 were either from the reported literature [62] or designed and purchased from Sangon Biotech.

GCLC shRNA-1: 5′-GCTAATGAGTCTGACCATTTT-3′;

GCLC shRNA-2: 5′-GTAGTATTCTGAACTACCTAA-3′;

SIRT2 shRNA-1: 5′-CAGCGCGTTTCTTCTCCTGTA-3′;

SIRT2 shRNA-2: 5′-CCTGCTCATCAACAAGGAGAA-3′.

Scrambled Control shRNA: 5′- GAGCGAGGGCGACTTAACCT -3′.

Lentivirus was packaged with plasmids psPAX2 and pMD2G in HEK293T cells, and used to infect target cells for at least 72 h.

### Vector construction and transfection

PCR-amplified human GCLC was cloned into p3xFLAG-CMV-10 or pGEX-5X-1; human SIRT2 was cloned into p3xFLAG-CMV-10, pcDNA6B-HA, or pET-28b; human SIRT1 and 3–7 were cloned into pcDNA6B-HA. SIRT2 and GCLC mutant constructs were generated with KOD Plus Mutagenesis Kit (KOD-401, TOYOBO). All expression constructs were validated by DNA sequencing. Lipomax reagent was used for transfection (LipoMax32012, Sudgen), and correct expression was validated by Western blotting.

### Co-immunoprecipitation (Co-IP) assay

Cells after indicated treatments were lysed in IP buffer (50 mM Tris-HCl, pH 7.5, 0.5% NP-40 (MF430, Mei5bio), 150 mM NaCl, 5 mM EDTA, 10% Glycerin) with protease inhibitor cocktail (B14001, Bimake) for 30 min at 4 °C, followed by the enrichment of target proteins using Flag-M2 beads (B23102, Bimake) or specific antibodies overnight at 4 °C. After washed with IP buffer for three times, and the binding complex proteins were eluted from the beads heated at 95 °C for 10 min in 1 × Laemmli sample buffer, and separated on sodium dodecyl sulfate-polyacrylamide gel electrophoresis (SDS-PAGE). Western blotting analysis was performed using indicated antibodies.

### GST pull-down assay

The GST-GCLC, GST, His-SIRT2 fusion protein were expressed in *E. coli* BL21 (DE3) cells and purified as described previously [63]. The GST-tagged proteins were first immobilized on GSH sepharose 4B (16100, Pierce) at 4 °C overnight, followed by incubation with His-SIRT2 at 4 °C for 4 h. The sepharose beads were then washed three times with lysis buffer, and boiling in 50 μL 2 × SDS loading buffer. The eluted samples were sujected to western blotting analysis using indicated antibodies.

### In vitro succinylation assay

Purified GCLC proteins were incubated with different concentrations of succinyl-CoA (S1129, Sigma-Aldrich) in TBS buffer (50 mM Tris-HCl [pH 7.5], 150 mM NaCl) at 37 °C for 4 h. The reaction was stopped by adding loading buffer and analyzed by western blotting analysis.

### In vitro desuccinylation assay

Hypersuccinylated GCLC proteins purified from HEK293T cells were incubated with purified SIRT2 in the presence or absence of 1 mmol/L NAD+ at 37 °C for 4 h with desuccinylation buffer (50 mmol/L Tris-HCl [pH 7.5], 150 mmol/L NaCl, 1 mmol/L MgCl_2_). The reaction was resolved on SDS-PAGE and analyzed by western blot analysis.

### Quantification of cell death and total cellular GSH level

Cells were seeded in 12-well plates for 24 h, followed by the indicated treatments. Cell death was stained with propidium iodide (PI) and analyzed by flow cytometry. The relative total GSH concentration in cells was assessed using the total GSH assay kit (S0052, Beyotime) according to the manufacturer’s instructions.

### Lipid peroxidation analysis using C11-BODIPY

Cells were seeded in 12-well plates. After indicated treatments, 2 μM C11-BODIPY 581/591 (27086, Cayman) was added and incubated for 0.5 h. After washed with PBS twice, cells were harvested by trypsin digestion and resuspended in PBS plus 5% FBS and analyzed by flow cytometry. 5000–10,000 cells were counted in each sample, and three independent experiments were performed.

### ROS analysis using DCFH-DA

Cells were seeded in 12-well plates for 24 h. After indicated treatments, 5 μM DCFH-DA (S0033S, Beyotime) was added for incubation of 0.5 h. After washed with PBS twice, cells were harvested by trypsinization and resuspended in PBS plus 5% FBS and analyzed by flow cytometry. 5000–10,000 cells were counted in each sample, and three independent experiments were performed.

### Animal experiment

Male C57BL/6 mice (8 weeks old) were purchased from Vital River Laboratory Animal Technology (Beijing, China). TBH (300 mg/kg) was intraperitoneal injected into mice for 3 h or 16 h. Liver tissues were harvested and MDA was measured using lipid peroxidation MDA assay kit (S0131S, Beyotime), total GSH level was measured using total GSH assay kit (S0052, Beyotime).

### Statistical analyses

GraphPad Prism 8 was used for all statistical analyses. The data were expressed as mean ± standard deviation (SD), from at least three independent experiments. A Student’s *t* test was used to compare two groups affected by a single variable. One-way ANOVA or two-way ANOVA with Turkey’s test was used to compare multiple data groups affected by one or two independent variables, respectively. *P*  <  0.05 was considered statistically significant.

## Supplementary information

Supplementary Information

Original Western blots

## Data Availability

All datasets generated for this study are included in the article/Supplementary Material.
