# Supplementary material for: GCLC desuccinylation regulated by oxidative stress protects human cancer cells from ferroptosis
Source: Cell Death Differ. 2025 Apr 5;32(9):1679–90. doi: 10.1038/s41418-025-01505-8 (PMC12432198; doi:10.1038/s41418-025-01505-8)
Supplement: Supplementary file 1 — Supplementary Information [file 41418_2025_1505_MOESM1_ESM.docx]

**Supplementary information**


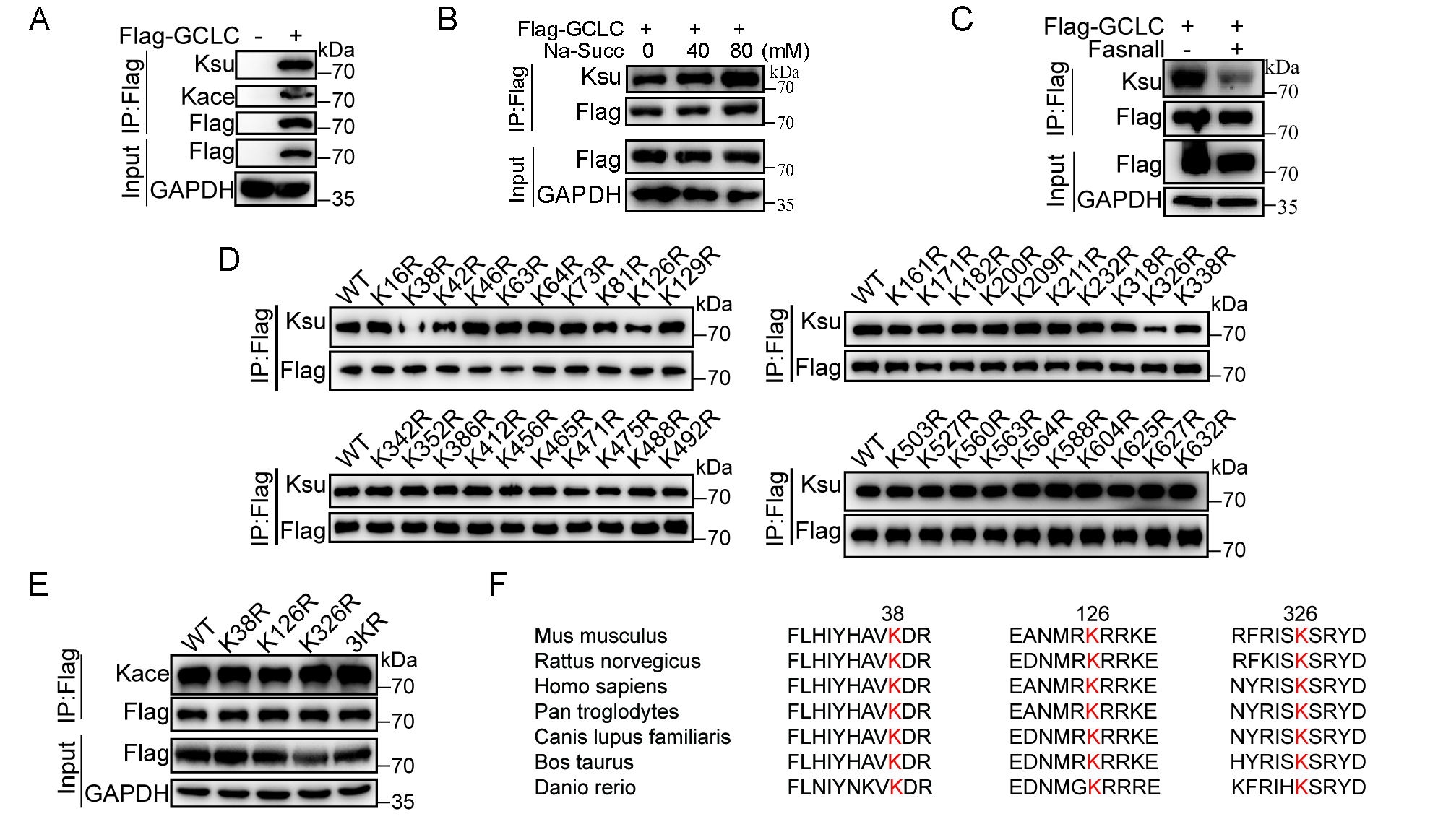


**Supplementary Fig. 1 Identification of GCLC succinylation sites.** **A** GCLC undergoes acetylation and succinylaiton modification. 293T cells were transfected with Flag-GCLC for 36 h, cell lysates were immunoprecipitated with anti-Flag and analyzed by western blotting with indicated antibodies. **B** Sodium succinate enhances the succinylation level of GCLC. HEK293T cells were transfected with Flag-GCLC for 36 h, then treated with different concentrations of sodium succinate for 24 h. Cell lysates were immunoprecipitated with anti-Flag and analyzed by western blotting with indicated antibodies. **C** Depletion of succinyl-CoA by Fasnall decreases the succinylation level of GCLC. HEK293T cells were transfected with Flag-GCLC for 36 h, then treated with 5 μM Fasnall for 24 h. Cell lysates were immunoprecipitated with anti-Flag and analyzed by western blotting with indicated antibodies. **D** Mapping the major lysine succinylation sites of GCLC. WT GCLC and indicated K to R mutantion constructs were transfected into 293T cells for 48 h, cell lysates were immunoprecipitated with anti-Flag antibody and analyzed by western blotting with indicated antibodies. **E** K38, K126, K326 are not the acetylation sites. GCLC WT and indicated K to R mutants constructs were transfected into 293T cells for 48 h, Cell lysates were immunoprecipitated with anti-Flag and analyzed by western blotting with indicated antibodies. **F** Alignment of multiple GCLC protein sequences across species.


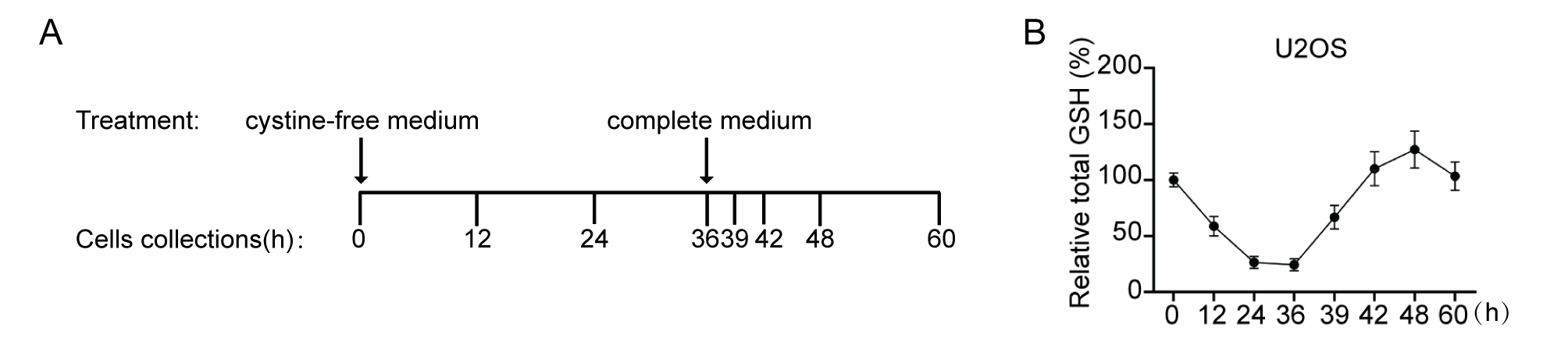


**Supplementary Fig. 2** **Synthesis rate of GSH in U2OS cells.** **A** Time line of the experiments, indicating the time points of treatment and cells collections. **B** Cellular GSH was measured at indicate times. The relative GSH level showed a linear increase between 36-42 hours, and the slope of this line represent the rate of GSH synthesis.


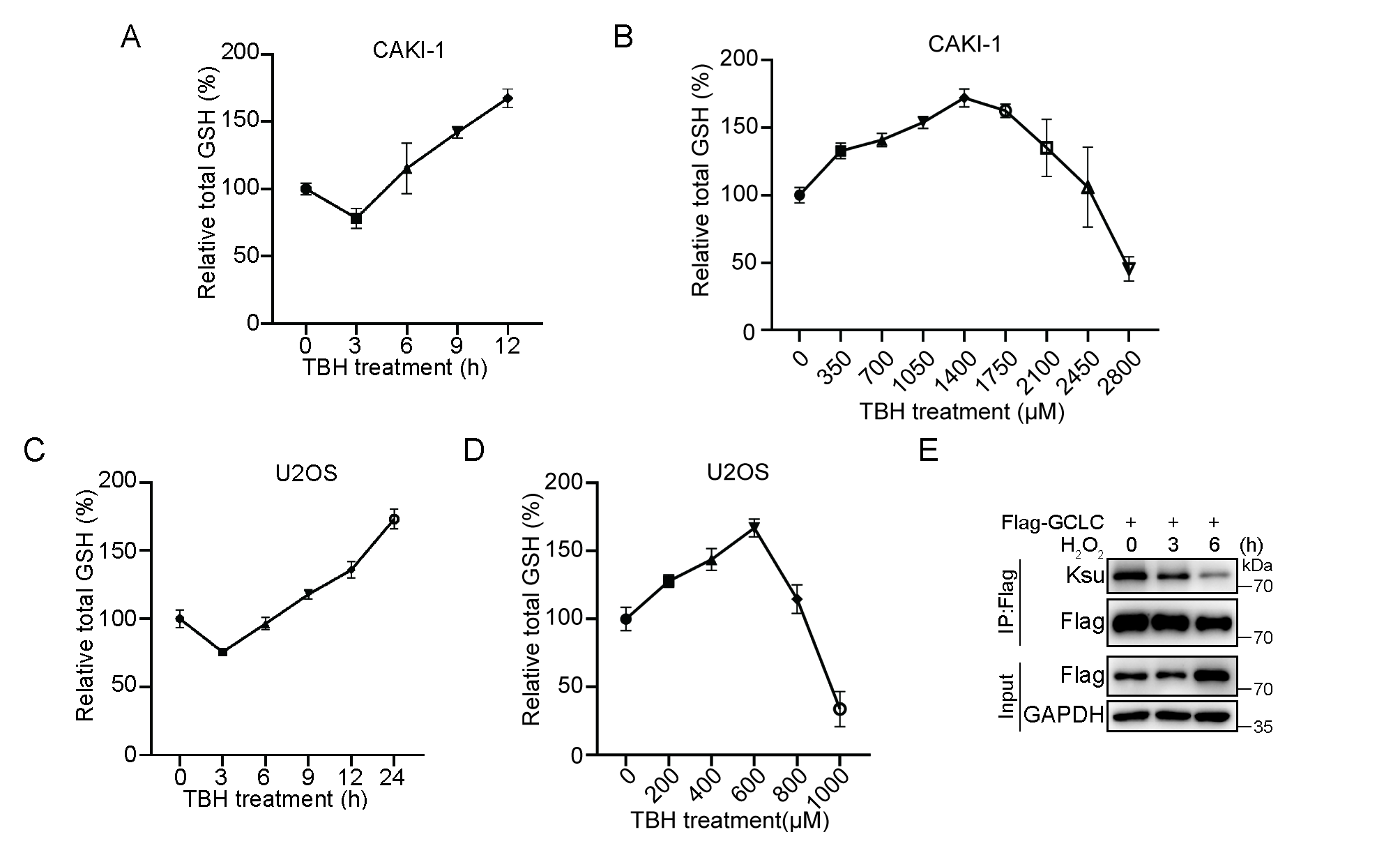


**Supplementary Fig. 3 Oxidative stress increases cellular GSH.** **A, B** TBH enhances cellular GSH in CAKI-1 cells. CAKI-1 cells were treated with 1400 μM TBH for different times, intracellular GSH level was measured (**A**). CAKI-1 cells were treated with different concentrations of TBH for 12 h, intracellular GSH level was measured (**B**). **C, D** TBH enhances cellular GSH in U2OS cells. U2OS cells were treated with 600 μM TBH for different time, intracellular GSH level was measured (**C**). U2OS cells were treated with different concentrations of TBH for 24 h, intracellular GSH level was measured (**D**). **E** H_2_O_2_ decreases GCLC succinylation in time-dependent manner. Flag-GCLC overexpressing U2OS cells were treated with 700μM H_2_O_2_ for different times. Cell lysates were immunoprecipitated with anti-Flag and analyzed by western blotting using indicated antibodies.


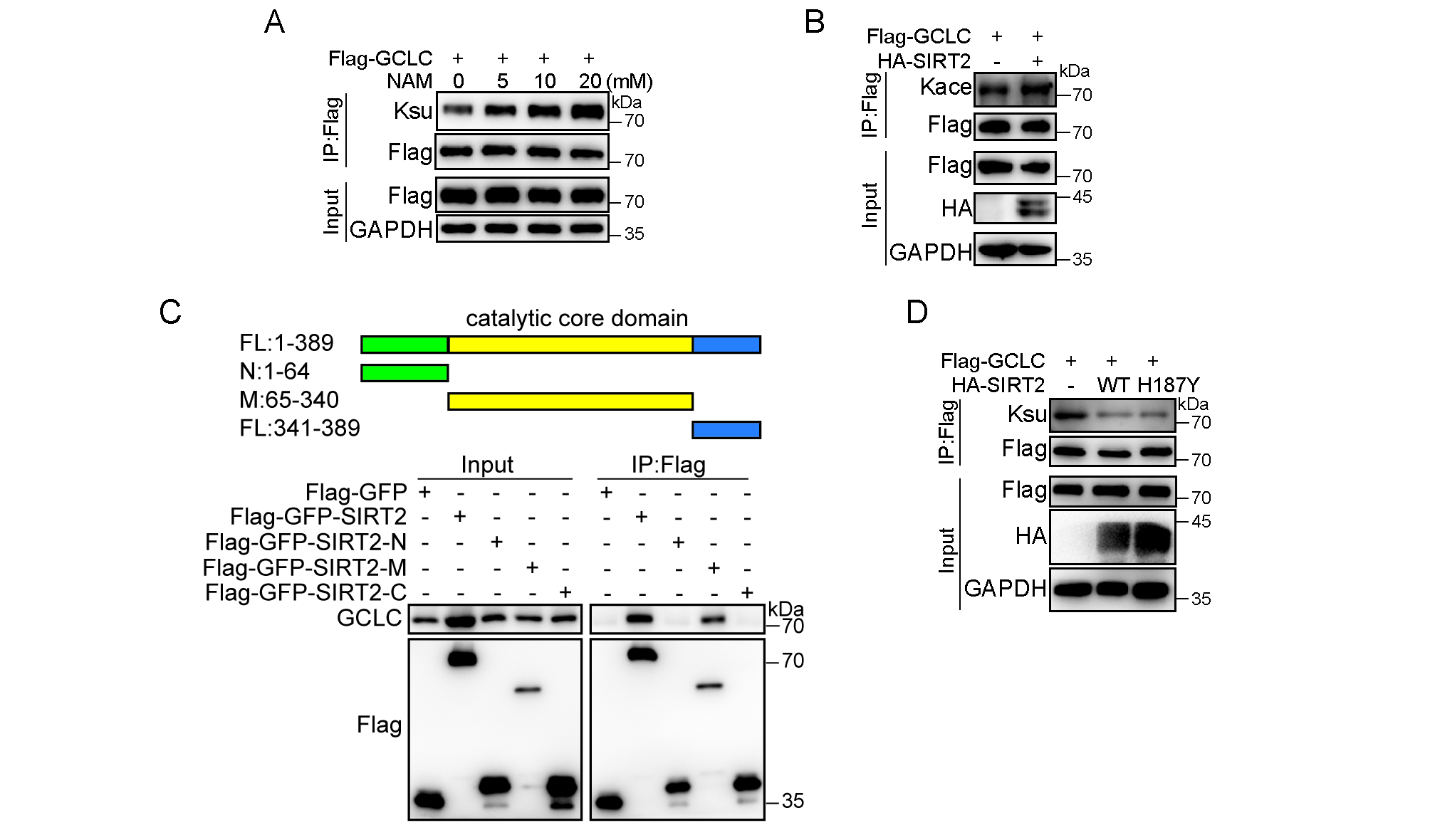


**Supplementary Fig. 4 GCLC succinulation is regulated by SIRT2 and interacts with the catalytic core domain of SIRT2. A** SIRT family inhibitor NAM enhances the succinylation of GCLC *in vivo*. 293T cells were transfected with Flag-GCLC for 36 h, then treated with different concentrations of NAM as indicated for 24 h, followed by immunoprecipitation with anti-Flag and western blotting analysis. **B** SIRT2 don’t affect GCLC acetylation. 293T cells were transfected with indicated expression constructs for 48 h, followed by immunoprecipitation with anti-Flag and western blotting analysis. **C** GCLC interacts with the catalytic domain of SIRT2. Schematic representation of SIRT2 and its mutants. 293T cells were transfected with indicated expression constructs for 48 h, followed by immunoprecipitation with anti-Flag and western blotting analysis. **D** SIRT2 acetylation inactive mutant desuccinylates GCLC *in vivo*. 293T cells were transfected with indicated expression constructs for 48 h, followed by immunoprecipitation with anti-Flag and western blotting analysis.


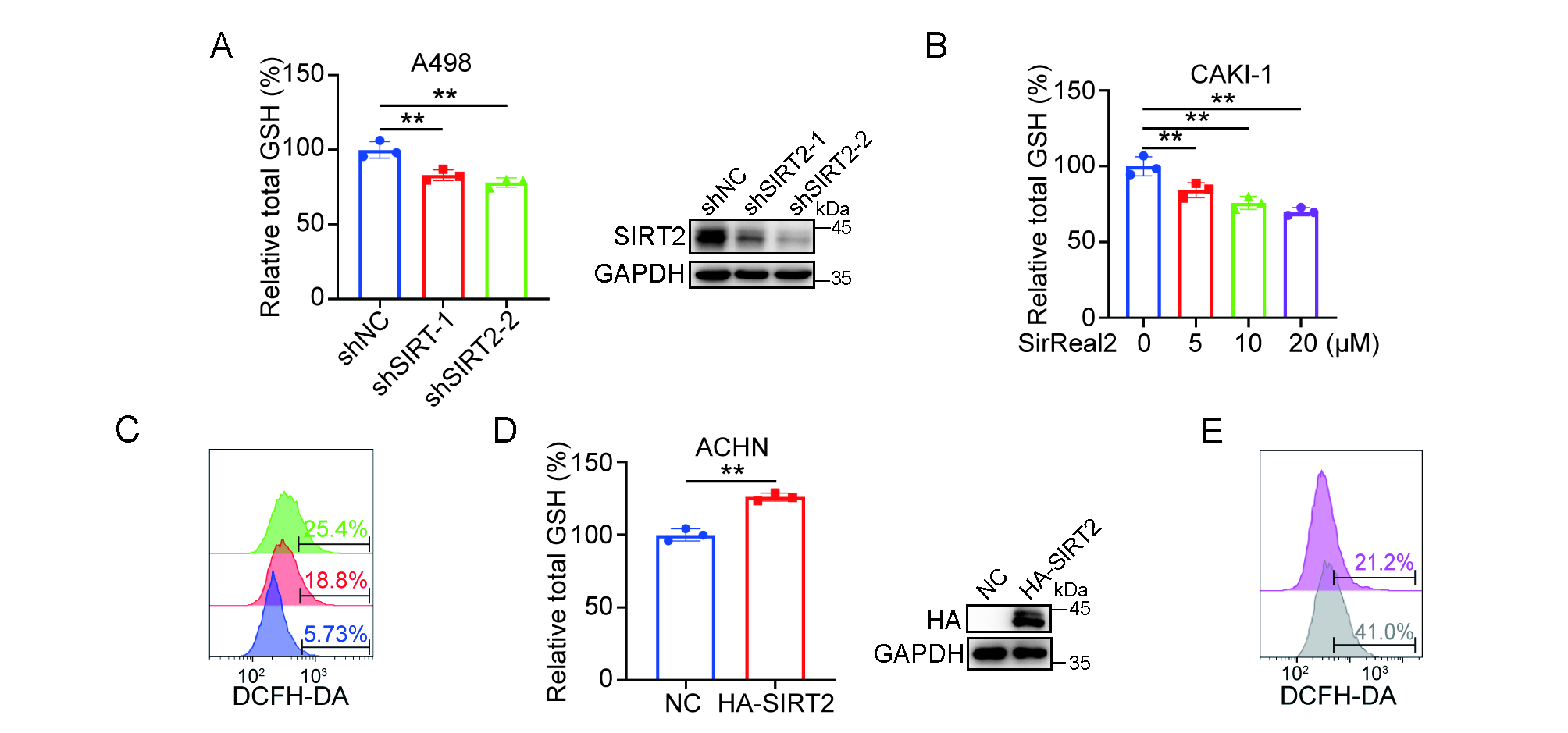


**Supplementary Fig. 5 SIRT2 regulates GSH synthesis. A** Knocking down of SIRT2 decreases cellular GSH level. A498 cells were transfected with indicated expression constructs for 72 h, intracellular GSH level and the expression of proteins were examined. **B** SirReal2 decreases cellular GSH level. CAKI-1 cells were treated with different concentrations of SirReal2 as indicated for 24 h, intracellular GSH level was measured. **C** Knocking down of SIRT2 increases cellular ROS level. CAKI-1 cells were transfected with indicated expression constructs for 72 h, intracellular ROS level was measured. **D** Overexpression of SIRT2 enhances cellular GSH level. ACHN cells were transfected with indicated expression constructs for 48 h, intracellular GSH level and the expression of proteins were examined. **E** Overexpression of SIRT2 decreases cellular ROS level. 786-O cells were transfected with indicated expression constructs for 48 h, intracellular ROS level was measured. Data were showed as mean ± standard error of mean (SEM) of at least three independent experiments. Statistical analysis by Student’s t test or one-way ANOVA. *P ≤ 0.05, **P ≤ 0.01.


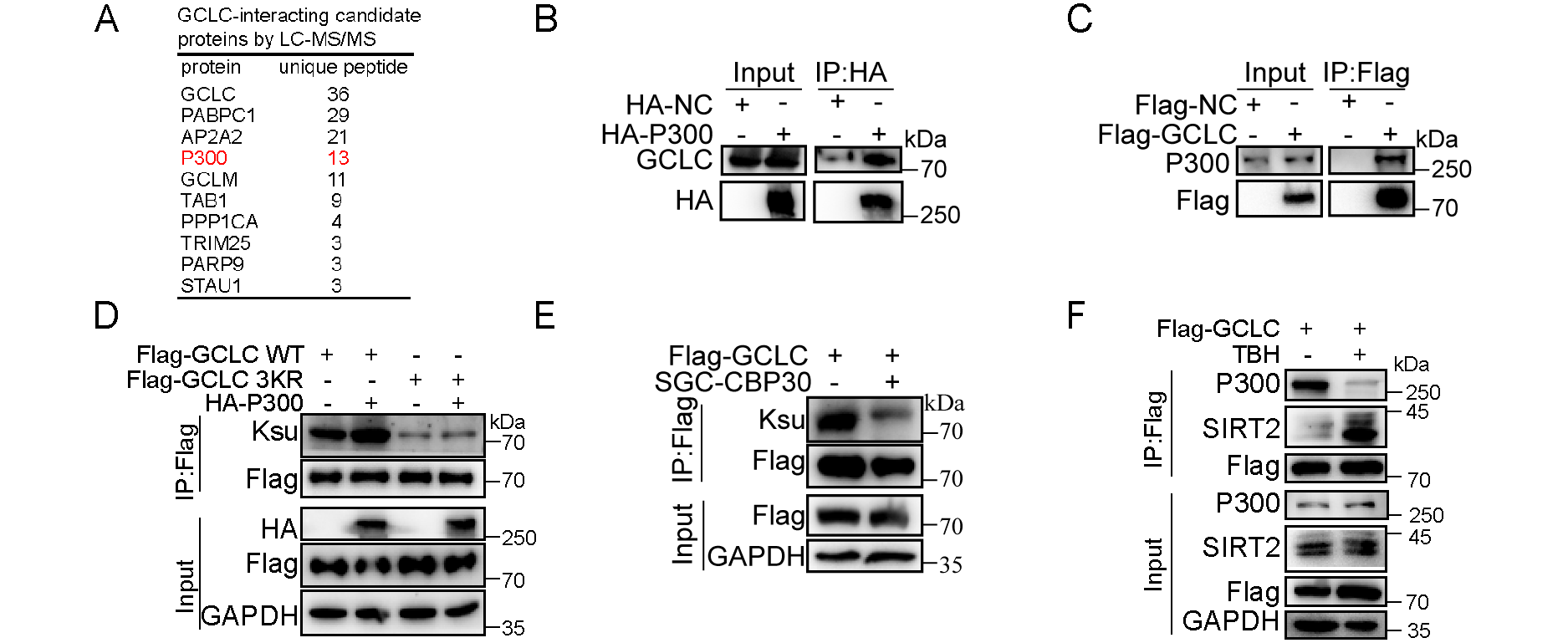


**Supplementary Fig. 6 P300 succinylates GCLC by interacting with GCLC. A** The list of GCLC-interacting partners. **B** HA-P300 interacts with endogenous GCLC. **C** Flag-GCLC interacts with endogenous P300. **D** P300 succinylates GCLC. HEK293T cells were transfected with indicated expression constructs for 48 h, followed by immunoprecipitation with anti-Flag and western blotting analysis. **E** P300 inhibitor SGC-CBP30 reduces the succinylation of GCLC. HEK293T cells were transfected with Flag-GCLC for 36 h, then treated with 10 μM SGC-CBP30 for 24 h. Cell lysates were immunoprecipitated with anti-Flag and analyzed by western blotting with indicated antibodies. **F** TBH decreases the interaction between P300 and GCLC. HEK293T cells were transfected with Flag-GCLC for 48 h, then treated with 700 μM TBH for 12 h. Cell lysates were immunoprecipitated with anti-Flag and analyzed by western blotting with indicated antibodies.


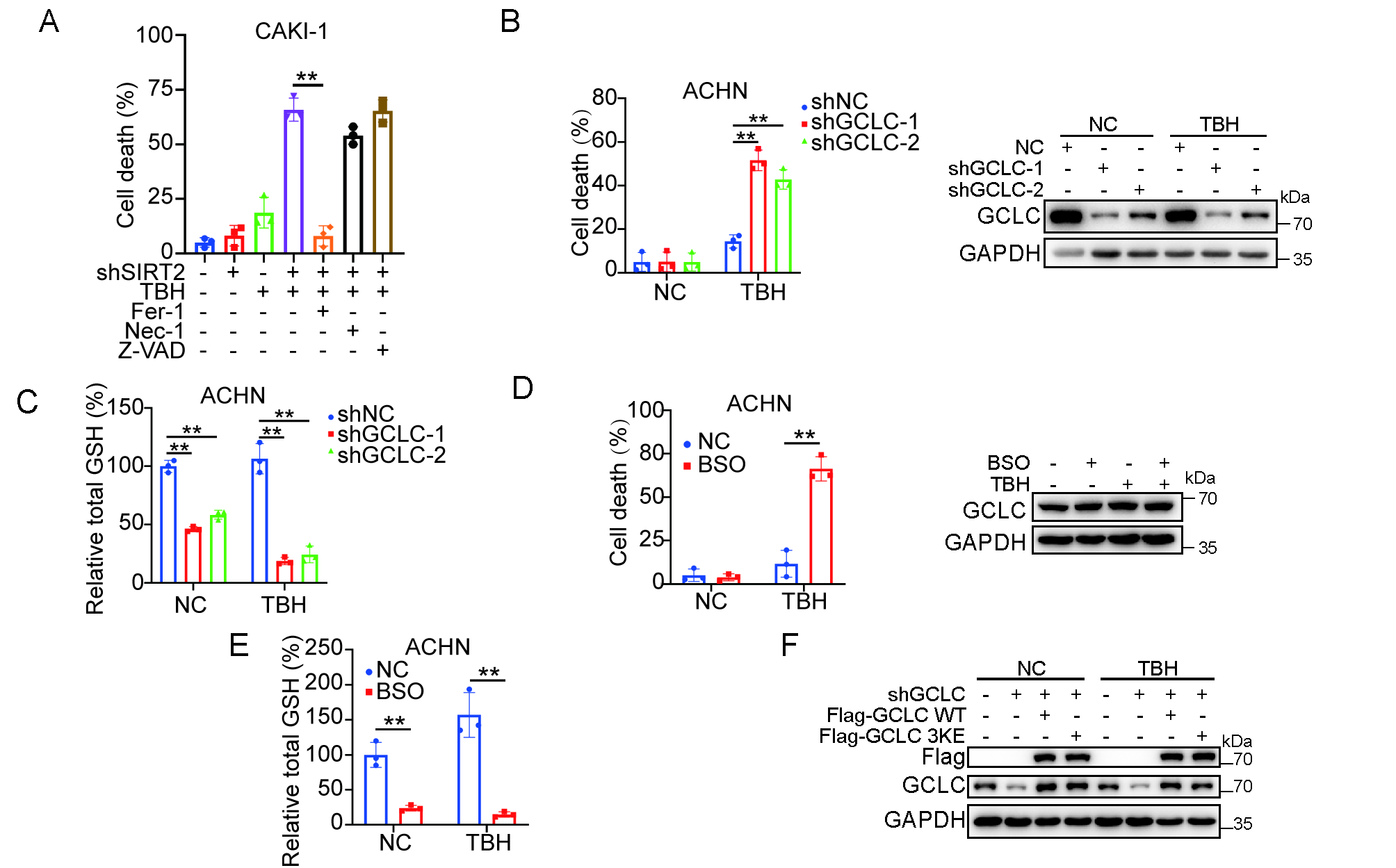


**Supplementary Fig. 7 Knocking down of GCLC enhances TBH induced ferroptosis.** **A** Ferroptosis inhibitor fer-1 inhibits TBH induced cell death. CAKI-1 cells were pretreated with fer-1, nec-1, z-vad for 6 h, then treated with 2400 µM TBH for 12h, and cell death was determined by PI staining coupled with flow cytometry. **B,** **C** Knocking down of GCLC enhances TBH-induced ferroptosis. Indicated cells were treated with 1000 µM TBH for 12h, and cell death was determined by PI staining coupled with flow cytometry (**B**), intracellular GSH level and the expression of proteins were examined (**C**). **D, E** GCLC inhibitor BSO enhances TBH-induced ferroptosis. ACHN cells were pretreated with 100 μM BSO for 12 h, then treated with 1000 µM TBH for 12h, and cell death was determined by PI staining coupled with flow cytometry (**D**), intracellular GSH level and the expression of proteins were examined (**E**). **F** The expression of proteins was assessed by western blotting. Data were showed as mean ± standard error of mean (SEM) of at least three independent experiments. Statistical analysis by two-way ANOVA. *P ≤ 0.05, **P ≤ 0.01.


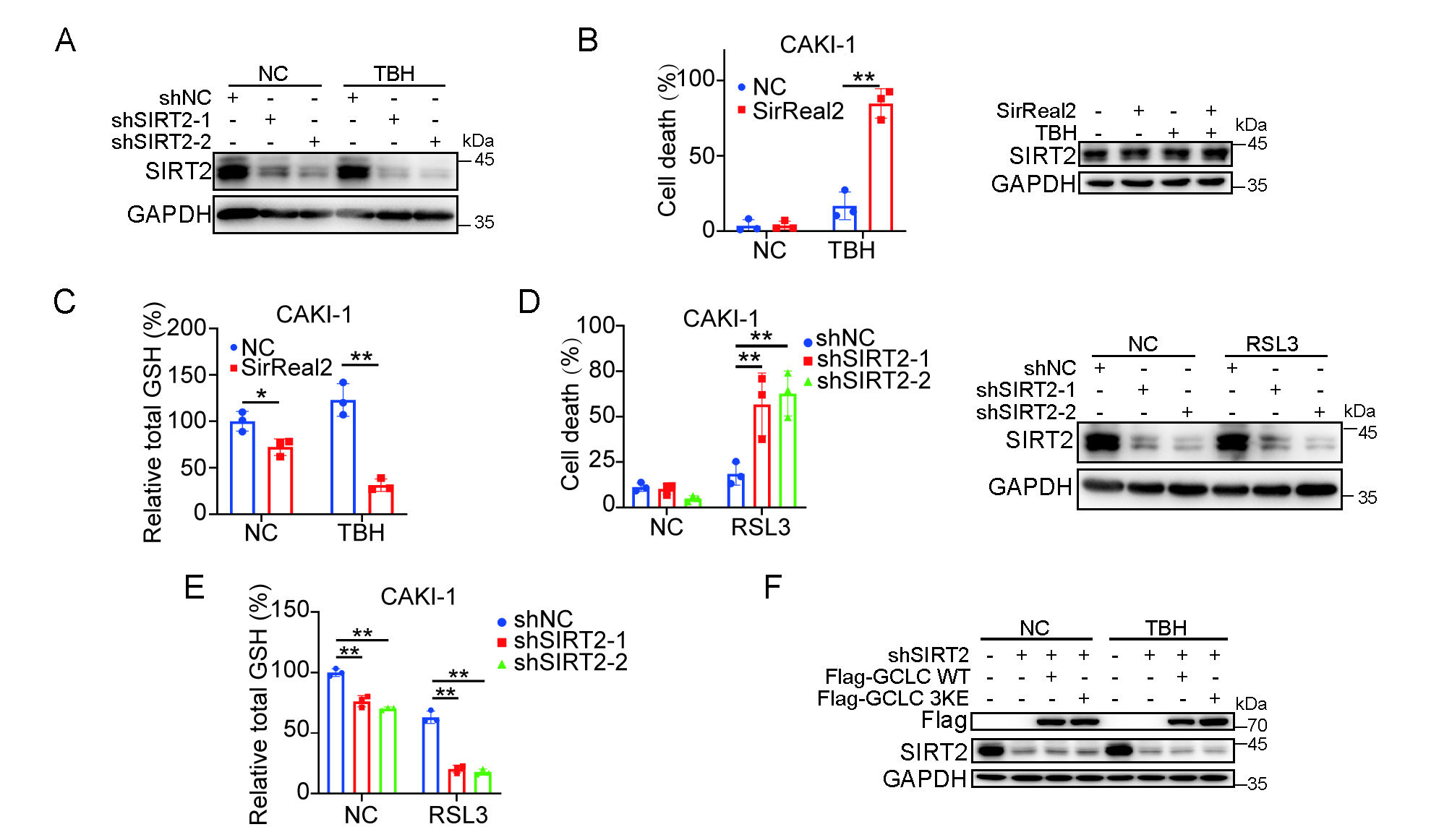


**Supplementary Fig. 8** **Knocking down of SIRT2 enhances TBH induced ferroptosis.** **A** The expression of proteins was assessed by western blotting. **B, C** SIRT2 inhibitor SirReal2 enhances TBH-induced ferroptosis. CAKI-1 cells were pretreated with 10 mM SirReal2 for 12 h, then treated with 2400 µM TBH for 12h, and cell death was determined by PI staining coupled with flow cytometry (**B**), intracellular GSH level and the expression of proteins were examined (**C**). **D, E** Knocking down of SIRT2 enhances TBH-induced ferroptosis. Indicated cells were treated with 0.8 µM RSL3 for 24h, and cell death was determined by PI staining coupled with flow cytometry (**D**), intracellular GSH level and the expression of proteins were examined (**E**). **F** The expression of proteins was assessed by western blotting. Data were showed as mean ± standard error of mean (SEM) of at least three independent experiments. Statistical analysis by one-way or two-way ANOVA. *P ≤ 0.05, **P ≤ 0.01.
